# Speciation in Coastal Basins Driven by Staggered Headwater Captures: Dispersal of a Species Complex, *Leporinus bahiensis*, as Revealed by Genome-wide SNP Data

**DOI:** 10.1093/sysbio/syad034

**Published:** 2023-06-01

**Authors:** Jorge L Ramirez, Carolina B Machado, Paulo Roberto Antunes de Mello Affonso, Pedro M Galetti

**Affiliations:** Facultad de Ciencias Biológicas, Universidad Nacional Mayor de San Marcos, Lima, Peru; Museo de Historia Natural de la Universidad Nacional Mayor de San Marcos, Lima, Peru; Departamento de Genética e Evolução, Universidade Federal de São Carlos, São Carlos, SP, Brazil; Departamento de Ciências Biológicas, Universidade Estadual do Sudoeste da Bahia, Jequié, BA, Brazil; Departamento de Genética e Evolução, Universidade Federal de São Carlos, São Carlos, SP, Brazil

**Keywords:** ABC, freshwater fishes, genotype by sequencing, neotropics, phylogeography

## Abstract

Past sea level changes and geological instability along watershed boundaries have largely influenced fish distribution across coastal basins, either by dispersal via palaeodrainages now submerged or by headwater captures, respectively. Accordingly, the South American Atlantic coast encompasses several small and isolated drainages that share a similar species composition, representing a suitable model to infer historical processes. *Leporinus bahiensis* is a freshwater fish species widespread along adjacent coastal basins over narrow continental shelf with no evidence of palaeodrainage connections at low sea level periods. Therefore, this study aimed to reconstruct its evolutionary history to infer the role of headwater captures in the dispersal process. To accomplish this, we employed molecular-level phylogenetic and population structure analyses based on Sanger sequences (5 genes) and genome-wide SNP data. Phylogenetic trees based on Sanger data were inconclusive, but SNPs data did support the monophyletic status of *L. bahiensis*. Both COI and SNP data revealed structured populations according to each hydrographic basin. Species delimitation analyses revealed from 3 (COI) to 5 (multilocus approach) MOTUs, corresponding to the sampled basins. An intricate biogeographic scenario was inferred and supported by Approximate Bayesian Computation (ABC) analysis. Specifically, a staggered pattern was revealed and characterized by sequential headwater captures from basins adjacent to upland drainages into small coastal basins at different periods. These headwater captures resulted in dispersal throughout contiguous coastal basins, followed by deep genetic divergence among lineages. To decipher such recent divergences, as herein represented by *L. bahiensis* populations, we used genome-wide SNPs data. Indeed, the combined use of genome-wide SNPs data and ABC method allowed us to reconstruct the evolutionary history and speciation of *L. bahiensis*. This framework might be useful in disentangling the diversification process in other neotropical fishes subject to a reticulate geological history.

Freshwater fish are restricted to aquatic habitats, and their dispersal modes depend on temporal connectivity between hydrographic systems. As such, their evolutionary history is largely correlated to the geological history of river basins (Hubert et al. 2007; Reis et al. 2016). Therefore, 2 major processes can affect fish distribution along coastal basins: 1) past sea level changes (Weitzman et al. 1988; De Bruyn et al. 2013; Dias et al. 2014; Thomaz et al. 2017) and 2) geological instability of the watershed that divides adjacent basins (Ihering 1898; Buckup 2011). In the former, periods of sea level retreats, particularly during the Pleistocene (i.e., Last Glacial Maximum), would create putative migration routes via palaeodrainages across the exposed continental shelf, allowing colonization of new areas. However, after sea level transgression, populations would become isolated, again resulting in interpopulation differentiation and, eventually, speciation (Buckup 2011; Dias et al. 2014). The role of palaeodrainages in dispersal and divergence of aquatic organisms has been amply reported (Abreu and Calliari 2005; Dias et al. 2014; Thomaz et al. 2015; Thomaz and Knowles 2018).

In contrast, some authors have argued that sea level fluctuations could not account for the presence of sister groups in both coastal and inland basins (Ribeiro 2006; Menezes et al. 2008; Buckup 2011). This distribution pattern could be explained by geological instability along watershed boundaries. In this case, headwater captures would play a key role in the distribution of sister taxa by introducing lineages from upland basins into coastal basins (Ribeiro 2006). This process could take place repeatedly at different times, thus increasing the diversity of coastal basins via merging (geodispersal) and further separation of geographic areas (vicariance) (Albert and Carvalho 2011). Therefore, geodispersal would promote biotic dispersal while vicariance would result in posterior biotic isolation. Once introduced into coastal basins, the variation in sea levels could then act as a secondary driver of dispersal along adjacent coastal basins (Buckup 2011; Thomaz et al. 2017; Thomaz and Knowles 2020).

Molecular studies have thrown some cold water on the role of palaeodrainages in dispersal and divergence of aquatic organisms. In particular, these studies have reported a significant divergence among isolated populations of fishes from coastal basins. Such divergence suggests longer periods of isolation than those expected under a scenario of dispersal across palaeodrainages during sea level retreats in the Pleistocene (Menezes et al. 2008; Lima et al. 2017; Ramirez et al. 2017b; Barreto et al. 2022). Accordingly, headwater captures have been consistently reported as the main process involved in dispersal and differentiation of lineages among coastal basins, particularly across the northeastern region of South America (Menezes et al. 2008; Lima et al. 2017, 2021; Souza et al. 2020; de Sousa et al. 2021). However, these studies are still reduced in number when compared to other Neotropical regions.

The South American Atlantic coast comprises several small and currently isolated drainages, and they are characterized by high levels of endemism and distinct biogeographic ecoregions (Buckup 2011; Lujan and Armbruster 2011). Most of these basins share a similar species composition, including representatives also found in inland basins, reflecting their historical interrelationships (Buckup 2011; Lujan and Armbruster 2011; Thomaz and Knowles 2018). *Leporinus bahiensis*Steindachner (1875) is one such fish species described for Atlantic coastal basins. It is widespread in several hydrographic systems of Bahia (northeastern Brazil), an area characterized by a narrow continental shelf with no evidence of submersed palaeodrainage connectivity between adjacent basins (Thomaz and Knowles 2018). Because of these features, *L. bahiensis* stands out as a good model to evaluate the role of headwater capture in dispersal and speciation processes along coastal basins without the influence of palaeodrainage connections at low sea level periods.

Therefore, in this study, we herein reconstructed the evolutionary history of *L. bahiensis* along its range based on molecular phylogenetics, population structure analyses and Approximate Bayesian Computation (ABC), using Sanger sequencing of 2 mitochondrial and 3 nuclear genes, as well as genome-wide SNP data obtained by high-throughput Genotype by sequencing (GBS). Taking into account the distribution of *L. bahiensis* across distinct and isolated basins, we raised 3 main questions: (i) is *L. bahiensis* a monophyletic group?; (ii) does the genetic structure among populations correspond to speciation events?; (iii) could sequential headwater captures explain the current distribution of lineages/populations along coastal basins?.”

## Material and Methods

### Sample Collection

**Figure 1.**
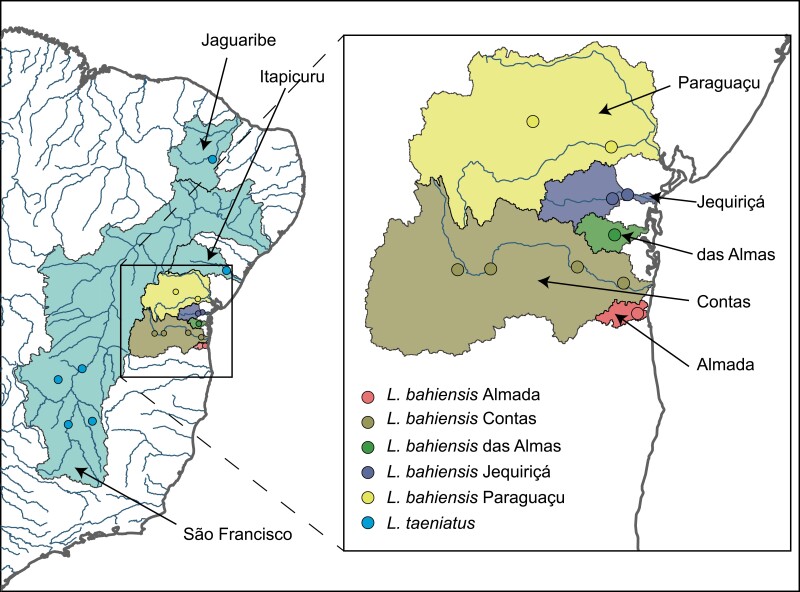
Map of Eastern Brazil showing hydrographic basins and collection sites of *L. bahiensis* and *L. taeniatus*.

Specimens of *L. bahiensis* were sampled from 5 coastal basins, including Paraguaçu (*n* = 6), Jequiriçá (*n* = 5), Almas (*n* = 4), Contas (*n* = 9), and Almada (*n* = 9), totaling 33 samples (Supplementary Table S1 and Fig. 1). Another 2 closely related *Leporinus* species (Ramirez 2015) were included for comparative analyses: *Leporinus taeniatus* Lütken 1875 from São Francisco, Itapicuru, and Jaguaribe basins and *Leporinus octofasciatus* Steindachner 1915 from Paraná basin (Supplementary Table S1). *Leporinus bahiensis* could be separated from all congeners, except *L. paranensis* from Upper Paraná, by having a dental formular of 3/4, 35 scales in the lateral line, 16 circumpeduncular scales, and 3 round dark blotches on lateral line supplemented by several brown bars across the dorsum (Steindachner 1875; Garavello and Britski 1987). Collection and transportation of specimens were carried out under permission (license #32215) provided by the Institute Chico Mendes de Conservação da Biodiversidade (ICMBio). Vouchers were deposited in recognized ichthyological collections (Supplementary Table S1).

All procedures were performed under ethical and legal recommendations as proposed by the Federal Council of Biologists, Resolution 301 (8 December 2012) and approved by the Ethics Committee of Utilization of Animals from the Universidade Estadual do Sudoeste da Bahia (CEUA/UESB, number 32/2013). Genetic heritage access was registered under number AAA03B9 at SisGen.

### DNA Extraction and Sanger Sequencing

Total DNA was isolated using DNeasy blood and tissue kit (Qiagen) following the manufacturer’s instructions. We sequenced the partial mitochondrial Cytochrome C Oxidase Subunit I (COI; 698 bp) and Cytochrome b (Cytb; 1110 bp) genes using AnosCOIF-AnosCOIR and AnosCytBF-AnosCytBR primers, respectively (Ramirez and Galetti 2015). The nuclear Myosin heavy chain 6 cardiac muscle alpha gene (Myh6, ~750 bp), recombination activating gene 1 (RAG1, ~1500 bp), and recombination activating gene 2 (RAG2, ~1100 bp) were sequenced using primers and protocols described elsewhere (Li and Ortí 2007; Li et al. 2007; Oliveira et al. 2011). All sequences were deposited in GenBank (Supplementary Table S1).

### Genotype By Sequencing Library and Sequencing

GBS libraries were constructed using commercial services (Ecomol Consultoria, Brazil). Total DNA (10 ng) was digested with PstI restriction enzyme and ligated with barcode adaptors. The libraries were purified, quantified, pooled and size-selected, following sequencing in the Illumina HiSeq 2500 using 1 × 100 (single read). Raw data were quality filtered, demultiplexed, and the output files were generated using the ipyrad v. 0.9.5 pipeline (Eaton and Overcast 2020). GBS data summary is shown in Supplementary Table S2 and available on Dryad via the link included in the Supplementary Material.

### Phylogenetic Analyses

Two data sets were used for phylogenetic reconstruction: 1) 2 mitochondrial and 3 nuclear genes and 2) SNPs from GBS. The first data set included genes successfully used to infer phylogenetic relationships within Anostomidae (Ramirez and Galetti 2015; Ramirez et al. 2016, 2017b, 2020). The final Sanger data set included 4 individuals of *L. bahiensis* and 3 specimens of *L. taeniatus* and 1 specimen of *L. octofasciatus*. These 3 species, together with *Leporinus paranensis*Garavello and Britski 1987, share a dental formula 3/4 and are closely related (Ramirez 2015). *Leporinus lacustris* Campos, 1945 was included as an outgroup since it is related to the *L. taeniatus* clade (Silva-Santos et al. 2018). Each gene was independently aligned using Clustal X2 (Larkin et al. 2007), and no gaps were observed. A maximum parsimony analysis for the concatenated sequences was performed in PAUP* 4.0b10 (Swofford 2003) with 1000 bootstrap replicates. A maximum likelihood (ML) tree was reconstructed using the concatenated alignments in RAxML for XSEDE (Stamatakis et al. 2008) on the CIPRES Science Gateway web server (Miller et al. 2010), using a mixed partition model, as determined by PartitionFinder (Lanfear et al. 2012), with a GTR+G substitution model and 1000 bootstrap replicates. A multilocus Bayesian species tree was estimated on *BEAST v2.6.3 (Heled and Drummond 2010), using K80+I, HKY+I, K80, K80, and JC substitution models for COI, Cytb, Myh6, RAG1, and RAG2, respectively, based on the Bayesian Information Criterion, using jModelTest 2 (Darriba et al. 2012). The mitochondrial genes were linked to share a single tree and were set to have an effective population size one-quarter that of the nuclear genes. We used an uncorrelated log-normal relaxed clock in all partitions and birth and death model as tree prior. The tree was calibrated using the clock rate from previous Anostomidae studies (Ramirez et al. 2020) as 7.8 × 10^−9^, 8.9 × 10^−9^, 5.3 × 10^−10^, 8 × 10^−10^, 8.3 × 10^−10^ mutations/site/year COI, Cytb, Myh6, RAG1, and RAG2, respectively. Markov Chain Monte Carlo (MCMC) was implemented using 100 million generations, sampling every 5000 runs and a 10% burn-in. Convergence of analyses and effective sample size (>200) were evaluated in Tracer v. 1.7.1 (Rambaut et al. 2018).

Phylogenetic analyses using the SNP data set encompassed 31 individuals (27 *L. bahiensis*, 3 *L. taeniatus*, and 1 *L. octofasciatus*). All trees were rooted using *L. octofasciatus* as the outgroup. A ML tree using RAxML for XSEDE (Stamatakis et al. 2008) was generated by assembling the loci present in at least 22 individuals, thus totaling 19,540 loci. A quartet-based coalescent phylogeny was inferred using Tetrad available in ipyrad v. 0.9.5 (Eaton and Overcast 2020) based on the SVDquartets algorithm (Chifman and Kubatko 2014) and optimized for SNPs data. In this case, only those loci present in all individuals (5728 bp) and a randomly sampled single SNP from each locus were used since the algorithm relies on multilocus unlinked SNPs. All possible quartets (subset of 4 samples) were sampled, and the support values were assessed using 1000 non-parametric bootstrap replicates.

### Genetic Structure Analyses

A COI haplotype network was calculated using Median-Joining (Bandelt et al. 1999) with POPART software (Leigh and Bryant 2015). To estimate the number of genetic clusters, a hierarchical Bayesian clustering of individuals was performed using the SNPs data set (27 individuals, 10,438 bp) in STRUCTURE (Pritchard et al. 2000), as implemented in ipyrad v. 0.9.5 (Eaton and Overcast 2020). We tested *k* = 2 to 6 populations and ran 10 replicates for each *K* with 100,000 MCMC and a burn-in of 5000. Replicates were aligned using CLUMPP (Jakobsson and Rosenberg 2007), and the best *K* was selected based on Δ*K* statistics (Evanno et al. 2005). Hierarchical analyses were performed on genetic clusters comprising more than 1 watershed to assess fine-scale patterns of population structure.

### Species Delimitation Analyses

Species delimitation analyses were carried out based on 2 data sets. The first corresponded to COI fragments, widely used to evaluate single-locus species delimitation (Roxo et al. 2015; Ramirez et al. 2017a, 2020; Hofmann et al. 2019), comprising 42 individuals that included 24 samples of *L. bahiensis*. For this marker, 3 methods of species delimitation were used: the general mixed Yule coalescent model with a single threshold (Pons et al. 2006), the Poisson tree processes (PTP) (Zhang et al. 2013), and the Bayesian implementation of the PTP model (bPTP) (Zhang et al. 2013). Because these methods require a phylogenetic tree as input, we generated an ultrametric tree using BEAST v. 2.6.3 (Bouckaert et al. 2014) with an HKY+I substitution model chosen by jModelTest2 (Darriba et al. 2012), a birth and death tree model, and an uncorrelated log-normal relaxed clock. Finally, all delimitation results were compared, and consensus Molecular Operational Taxonomic Units (MOTUs) were generated using the SPdel pipeline (https://github.com/jolobito/SPdel) (Ramirez et al. 2020). Additionally, COI intra- and inter-group genetic distances were estimated using the K2P model in the same SPdel pipeline.

In addition, the SNPs data set was selected for multilocus species delimitation using Bayes Factor Delimitation (BFD) (Leaché et al. 2014) and BPP v. 4.38 (Yang and Rannala 2010). For both algorithms, a maximum of 4 individuals for each basin was included, with no missing data, owing to computational constraints. Therefore, the data set included 17 samples of *L. bahiensis* and one each of *L. taeniatus* and *L. octofasciatus*. For BFD, we estimated a coalescent-based species tree using SNAPP (Bryant et al. 2012) to test 5 alternative species delimitation models, separating the populations of *L. bahiensis* according to basin (Table S3). We assigned a Gamma distribution to Lambda prior (Alpha = 2.5, Beta = 2.0, and Mean = 5) and set Theta = 0.01 (Alpha = 1.0, Beta = 100, and Lambda = 5). SNAPP analyses were performed in BEAST v. 2.6.3 (Bouckaert et al. 2014) with a path sampling of 24 steps and 200,000 MCMC generations, sampling every 1000 generations. Marginal likelihood (ML) estimates for each model were ranked using BFD, and BFD was calculated based on twice the difference in marginal likelihood estimates for 2 competing methods (Leaché et al. 2014). For BPP, we conducted species delimitation based on a guide tree (Yang and Rannala 2010; Rannala and Yang 2013), using options speciesdelimitation = 1 and speciestree = 0. The fixed tree was based on the Tetrad result, and the putative species were designated by basin. The analyses were performed with 500 loci randomly sampled and 2 replicate runs under different seeds. Prior θ (genetic diversity) and τ (root age) were calculated using the Minimalist BPP app found at https://brannala.github.io/bpps/. We set the inverse-gamma prior θ to the average pairwise difference among sequences within each population, IG (3, 0.12), and tested 2 alternative τ corresponding to the maximum pairwise differences: 1) all *L. bahiensis* populations refer to distinct species, IG (3, 0.0014) or 2) a single species, IG (3, 0.0018). A range of fine-tuning algorithm parameters was used: algorithm 0 with ε = 1, 2, and 5 and algorithm 1 with α = 1, 1.5, and 2 and *m* = 0.5, 1, and 2. For all runs, 200,000 MCMC generations were sampled every 2 generations with a burn-in of 20,000.

### Dispersal Routes Assessment Using ABC approach

**Figure 2.**
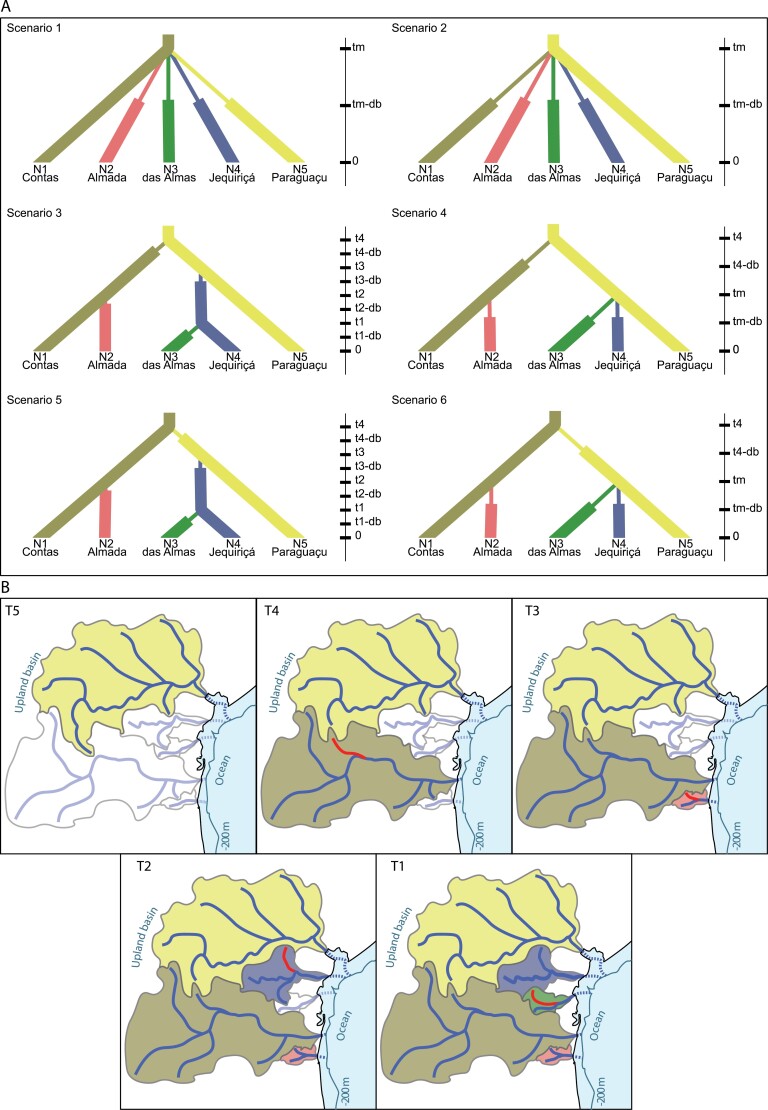
Models explaining the staggered dispersal processes. A) Alternative phylogenetic scenarios to be tested through an approximate Bayesian computation approach (ABC). N: effective population size; t: time given in generations, db: time during the bottleneck event. Width on the tree branch indicates changes in effective population size. Vertical bar indicates time backwards. B) Geographic scenario showing the staggered dispersal by sequential headwater captures as hypothesized in scenario 3 (scenario chosen by the ABC analysis). River networks are illustrative. T4–T0, time steps, from past to present. Shaded regions are occupied basin. The captured river in each time is in red. In blue, coastline during the low sea level period. Dashed rivers are palaeodrainages now submerged, showing no connection between them.

To understand the role of geodispersal events in the diversification of *L. bahiensis*, we tested alternative demographic scenarios using the ABC approach with DIYABC v2.1.0 software (Cornuet et al. 2014). Evolutionary scenarios were based on phylogenetic reconstruction analyses, placing Paraguaçu or Contas basin as the source population (Fig. 2) since both basins share boundaries with the São Francisco basin where the sister species (*L. taeniatus*) is distributed (Fig. 1). Scenarios 1 and 2 considered a unique event of divergence among all sampled basins. This could be expected when dispersal is driven by a single palaeodrainage connection during a period of sea level retreats (Fig. 2). Scenarios 4 and 6 considered an initial dispersal driven by headwater capture from Paraguaçu to Contas or vice versa, followed by a subsequent and simultaneous dispersal event among daughter populations by a palaeodrainage connection during a period of sea level retreats (Fig. 2). Scenarios 3 and 5 correspond to a staggered pattern in which the initial source population varies (Fig. 2). In this case, dispersal occurred from basins adjacent to upland drainages to small coastal basins by consecutive headwater captures at different times.

In all the above-described scenarios, an increase in effective population size (N_e_) of daughter populations after initial geodispersal was considered (Machado et al. 2018). Details about the tested demographic and time priors are provided in the Supplementary Table S4.

We simulated 1 million data sets for each scenario. To check the likelihood that both scenarios and prior distributions of parameters could produce a simulated data set close to the observed data set, we performed a pre-evaluation using Principal Components Analysis. The posterior probabilities of alternative scenarios were estimated by polychotomous logistic regression analysis, considering 1% of the total simulated data set closest to the observed data (Cornuet et al. 2008). The most probable scenario was then determined based on the highest and non-overlapped values of posterior probability within a 95% confidence interval (CI).

### Estimates of Divergence Times

DIYABC software does not require mutation model parameterization in SNPs data sets. However, this would result in the absence of any calibration brought by priors on mutation parameters. Therefore, we used a composite-likelihood method based on the site frequency spectrum (SFS) implemented in FASTSIMCOAL2 (Excoffier et al. 2013, 2021) to estimate divergence times between lineages according to the scenario obtained by ABC. The FASTSIMCOAL2 approach allowed us to simulate a scenario whereby 2 populations diverged and then estimate the time divergence by using a mutation rate and the effective population size of 1 population as reference. The SFS was constructed from the variant call format file without missing data and calculated by each pair of basins using easySFS (https://github.com/isaacovercast/easySFS). FASTIMCOAL2 analysis was performed using “removeZeroSFS” by assuming no migration between populations after splitting, as expected in a scenario of headwater capture. Then N_e_ of 1 of the 2 populations was calculated from nucleotide diversity, π, of fixed and variable sites, and a mutation rate (μ) of 2.24 × 10^−8^ was chosen (Thomaz et al. 2017). For each pair of basins, 40 replicates were run with 100,000–250,000 simulations, a stopping criterion of 0.001, and 10–40 expectation conditional maximization cycles for the calculation of composite likelihood. The 95% CIs of parameter estimates, including divergence time, were calculated from 100 parametric bootstrap replicates. Each replicate used a simulated SFS with the same number of individuals, loci and parameters from the maximum composite likelihood estimates to re-estimate parameters.

## Results

### Phylogenetic Analysis

**Figure 3.**
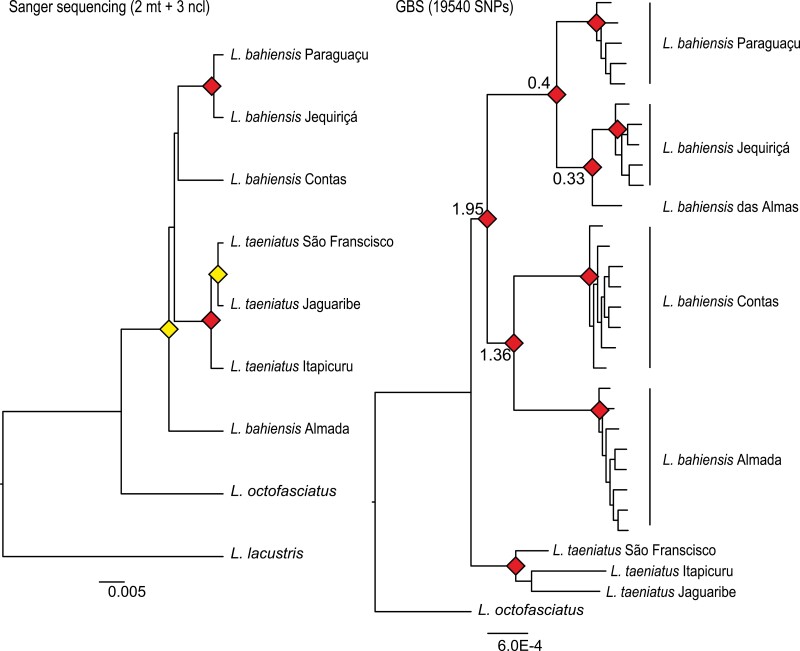
Phylogenetic trees of *L. bahiensis* and related species. Left: Bayesian species tree generated using 5 genes (approx. 4900 bp). Red diamonds indicate nodes with support values above 0.9 of posterior probability in Bayesian inference and bootstrap higher than 75% in MP and ML analyses. Yellow diamonds indicate high support values in 2 out of the 3 methods. Right: ML tree generated using SNPs data from GBS. Red diamonds indicate nodes with bootstrap values for ML and quartet analyses higher than 75%. Numbers in node indicate divergence time estimated using FASTSIMCOAL, in millions of years. Scale bar indicates nucleotide substitution per site.

The aligned sequences of COI, CytB, Myh6, RAG1 and RAG2 included 595, 1005, 733, 1160, and 1023 bp, and 30, 33, 3, 4, and 3 parsimony-informative sites, respectively. No gaps were observed in the alignments. All phylogenetic trees recovered the same topology, supporting a well-defined clade per basin, but the monophyletic status of *L. bahiensis* was not supported. Instead, the phylogenetic trees based on mitochondrial and nuclear genes recovered *L. bahiensis* and *L. taeniatus* as paraphyletic (Fig. 3, left, Supplementary Fig. S1).

On the other hand, the phylogenetic trees based on SNPs recovered *L. bahiensis* as a monophyletic group divided into highly supported subclades from each coastal basin (Fig. 3, right), placing *L. taeniatus* as a sister group of *L. bahiensis*. Two reciprocally monophyletic subclades were observed within *L. bahiensis*, comprising the samples from Paraguaçu, Jequiriçá, and Almas basins and another cluster that included populations from Contas and Almada basins.

### Genetic Structure of Leporinus bahiensis

**Figure 4.**
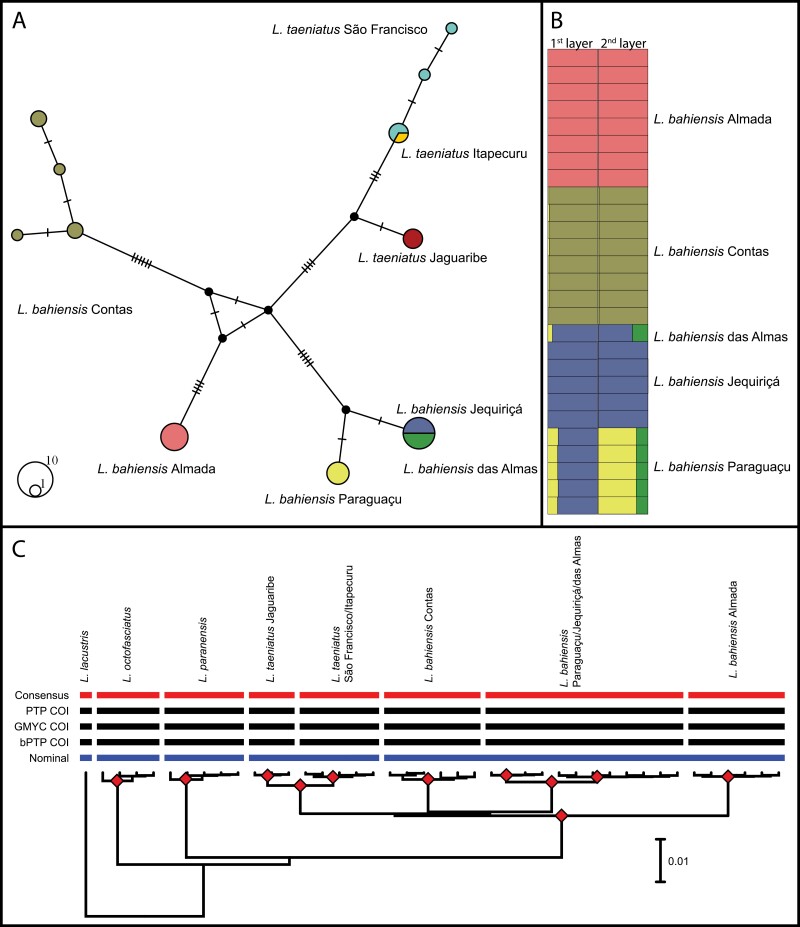
Genetic structure of *L. bahiensis*. A) Median-joining (MJ) haplotype network using COI data. Small dark dots indicate missing or unsampled haplotypes. B) STRUCTURE analysis based on SNPs data. The first layer included all individuals (*K* = 4), while the second layer encompassed only the specimens from Almas/Jequiriçá/Paraguaçu (*K* = 3). Vertical bars indicate individual membership coefficient. C) Bayesian tree showing the clustering of MOTUs obtained by species delimitation analyses based on COI. Blue line represents nominal taxonomy, and red line represents consensus MOTUs. Red diamonds indicate nodes with support values higher than 0.9 of posterior probability. Scale bar indicates nucleotide substitutions per site.

Eight COI haplotypes described in *L. bahiensis* (Fig. 4a) were distributed according to each basin, except for samples from Jequiriçá and Almas which shared the same haplotype.

Structure analysis revealed *K* = 4 with the highest Δ*K*, splitting *L. bahiensis* into 3 distinct clusters, Almada, Contas and a third one joining the samples from Paraguaçu, Jequiriçá and Almas, along with the signal of an admixture from a fourth and unsampled population in Paraguaçu and Almas (Fig. 4b, Supplementary Table S5). A hierarchical analysis within the latter cluster found *K* = 3 with the highest Δ*K*. Accordingly, Paraguaçu was separated from Jequiriçá and Almas, and it remained as a single group (Fig. 4b, Supplementary Table S5). The admixture signal from the unsampled population was once again suggested for the populations from Paraguaçu and Almas.

### Species Delimitation

The final aligned COI sequences resulted in 619 characters with 51 parsimony-informative sites. All single-gene species delimitation analyses invariably recovered 8 molecular units, regarded as consensus MOTUs, out of which 3 were related to *L. bahiensis* (Fig. 4c). These MOTUs are correlated with basins since most of them encompassed a unique MOTU, except for the populations from Paraguaçu/Jequiriçá/Almas that were clustered in a single MOTU. Genetic distances for both nominal species and consensus MOTUs are shown in Supplementary Table S6. The maximum intraspecific distance of *L. bahiensis* was 2.65%, while the maximum intra-MOTU distance was 0.49%, and the minimum inter-MOTU was 0.65%.

For the SNPs data set, we tested 5 species delimitation scenarios using BFD coalescent delimitation. The best-supported model comprised 7 lineages, 5 of which belonged to *L. bahiensis*, according to BFD (Supplementary Table S3). This model decisively supported the division of *L. bahiensis* according to basin. BPP delimitation also inferred 7 potential species with the lowest τ values for all tested priors (posterior probabilities > 0.9). This model also divided *L. bahiensis* into 5 distinct units. However, considering the highest τ prior, 5 of the 12 tests suggested the presence of 6 species (posterior probabilities lower than 0.9) joining Jequiriçá and Almas populations, but with low support values. The remaining tests supported the model that included 7 species (posterior probabilities > 0.9) (Supplementary Table S7).

### Dispersal Routes Assessment Using ABC

Demographic scenario 3 (Fig. 2) had the highest posterior probability using raw summary statistics (posterior probability = 1.0; 95% CI: 1.0–1.0), suggesting Paraguaçu basin as a source for 2 independent dispersal events towards Contas and Jequiriçá basins. Afterwards, the specimens from Contas would have colonized Almada basin, while another dispersal event was inferred from Jequiriçá to Almas basin. The confidence estimated for this scenario based on the logistic approach revealed a low posterior error rate (0.074), confirming the reliability of this analysis. As shown in Supplementary Table S8, most tested parameters presented low RMAE values (<0.2), except for 5 [N_Contas_ (N1), N_Almada_ (N2), N_b_Almada_ (N2b), N_b_das Almas_ (N5b), and T_bot_ (db)], indicating that the estimates are trustworthy (Supplementary Table S8; Cornuet et al. 2008). These results squarely place scenario 3 as the most appropriate model to explain the current genetic subdivision in *L. bahiensis*.

### Estimates of Divergence Times

FASTSIMCOAL2 divergence time estimates using the best model obtained by ABC analysis dated the first dispersal event from Paraguaçu to Contas around 1.95 Ma (95% CI: 1.94–2.1) (Fig. 3 right). The second dispersal event (Contas to Almada) took place about 1.36 Ma (95% CI: 1.36–1.47), while the dispersal from Paraguaçu to Jequiriçá occurred about 0.4 Ma (95% C.I. 0.39–0.43), followed by a dispersal event from the latter to Almas basin near 0.33 Ma (95% CI: 0.34–0.37).

## Discussion

The specimens morphologically recognized as *L. bahiensis* have been sampled in distinct coastal basins along the state of Bahia, northeastern Brazil. Surprisingly, the phylogenetic trees inferred from nuclear and mitochondrial markers, successfully used in studies of Anostomidae (Ramirez and Galetti 2015; Ramirez et al. 2016, 2017b, 2020), failed to recover the monophyly of *L. bahiensis* (Fig. 3). In fact, populations of *L. taeniatus* were placed as a subgroup within the cluster of *L. bahiensis* in spite of their differences in morphology and range. *L. taeniatus*, distributed in São Francisco and other rivers in northeastern Brazil (Garavello and Santos 2009), is characterized by a continuous narrow dark-brown stripe over a lateral line (Santos and Jégu 1996), while *L. bahiensis* is restricted to a few coastal basins and presents 3 large-rounded blotches along the midlateral line.

On the other hand, the SNPs data set resolved the phylogenetic relationships between both closely related species, supporting their reciprocal monophyletic status (Fig. 3), probably because genomic approaches, such as GBS, provided a larger amount of high-throughput sequencing data. Actually, the monophyletic signal, which is based on multiple loci (e.g., Sanger sequencing), requires longer periods of divergence (Rosenberg 2003; Knowles and Carstens 2007) and is, therefore, limited to the inference of evolutionary relationships in groups of rapid initial divergence. Different processes like incomplete lineage sorting and ancestral polymorphism can hinder phylogenetic reconstruction based on sequencing of a few loci, especially in cases of recent speciation (Knowles and Carstens 2007).

On the other hand, the genetic structure of *L. bahiensis* based on either COI or SNPs data usually supported the discrimination of populations according to each river basin (Fig. 4), except for samples from Jequiriçá and Almas basins that shared the same COI haplotype. Furthermore, the number of mutation steps between Jequiriçá/Almas and Paraguaçu samples was lower than that observed between other populations of *L. bahiensis*. Since these coastal basins have no evidence of past connections via palaeodrainages during low sea level periods (Thomaz and Knowles 2018), the genetic structure herein reported likely refers to allopatric processes.

When the structured lineages observed in *L. bahiensis* were tested for the putative presence of MOTUs using COI data, a total of 3 consensus MOTUs was obtained, corresponding, in part, to the sampled basins. Again, the only exception was observed in the samples from Jequiriçá/Almas/Paraguaçu, recovered as a single MOTU. It should be pointed out that the lowest genetic divergence in MOTUS of *L. bahiensis* based on COI was relatively high (1.71–1.89%) in comparison to previous reports in Anostomidae (Ramirez and Galetti 2015; Ramirez et al. 2017a, 2020).

In contrast, multilocus (SNPs) species delimitation analyses invariably indicated 5 MOTUS within *L. bahiensis*, each one corresponding to a single basin. These results reinforce the idea that multilocus analyses usually show higher resolution power than the single-locus species delimitation approach (Liu et al. 2017), especially when considering very recent events, such as that inferred among samples from Paraguaçu, Jequiriçá and Almas (<400 Kya). Nonetheless, following a conservative perspective, we suggest at least 3 MOTUs within *L. bahiensis*, as represented by the lineages from Almada, Contas, and Paraguaçu/Jequiriçá/Almas basins. This calls for a thorough and integrative taxonomic reevaluation to determine species status of these MOTUs. Moreover, the description of *L. bahiensis* should be reevaluated with emphasis on the definition of type locality since syntypes have been lost (Garavello and Britski 2003).

Results based on the ABC approach revealed that the diversification processes among populations of *L. bahiensis* could not be explained by simultaneous dispersal events via submersed palaeodrainages in Pleistocene, thus differing from other biogeographic reports in Neotropical freshwater fish (e.g., Hirschmann et al. 2015; Thomaz et al. 2015; Abreu et al. 2020; Thomaz and Knowles 2020). Indeed, the northeastern coast of Brazil, site of the present study, presents a relatively narrow continental shelf that would restrain the dispersal of populations by the lack of submersed palaeodrainages connecting coastal basins (Thomaz and Knowles 2018).

Thus, the best model to explain the present results would comprise a staggered pattern characterized by sequential events of headwater capture at different periods among basins. This inference is supported by previous reports, including distinct fish taxa from the sampled region, revealing the key role of headwater captures in the dispersal and genetic divergence among currently isolated populations, even before sea level changes in late Pleistocene (Menezes et al. 2008; Lima et al. 2017; Ramirez et al. 2017b; de Sousa et al. 2021; Barreto et al. 2022). In several cases, the remarkable genetic differentiation among populations of a single nominal taxon could only be explained by a complex network of headwater captures followed by isolation of drainages (Souza et al. 2020; de Sousa et al. 2021). Accordingly, this study reinforces the importance of headwater captures to the dispersal and further differentiation of freshwater fish populations along the South American Atlantic coast (Lima et al. 2017), as particularly validated through the ABC approach combined with a genome-wide SNPs data set (Fig. 2).

Finally, we can conclude that different approaches are required to overcome the difficulties in studying the evolutionary history of species with recent divergence, such as that observed in *L. bahiensis*. In particular, genome-wide SNPs data might be decisive in resolving cases where other markers (e.g., partial gene sequences) fail, as clearly demonstrated in the present phylogenetic reconstruction and species delimitation analyses. *Leporinus bahiensis* is recognized as a species complex composed of at least 3 MOTUs. This calls for a reexamination of its taxonomic status. Furthermore, combining the ABC approach with genome-wide SNPs data proved to be effective in reconstructing complex biogeographic scenarios. Going forward, this will help us understand speciation in other highly diverse groups like Neotropical fishes.

## Supplementary Material

Data available from the Dryad Digital Repository: http://dx.doi.org/10.5061/dryad.0zpc8670j.
